# Physiological and Biochemical Changes in *Brassica juncea* Plants under Cd-Induced Stress

**DOI:** 10.1155/2014/726070

**Published:** 2014-07-15

**Authors:** Dhriti Kapoor, Satwinderjeet Kaur, Renu Bhardwaj

**Affiliations:** Department of Botanical and Environmental Sciences, Guru Nanak Dev University, Amritsar, Punjab 143005, India

## Abstract

Plants of* Brassica juncea *L. var. RLC-1 were exposed for 30 days to different concentrations (0, 0.2, 0.4, and 0.6 mM) of cadmium (Cd) to analyze the Cd uptake, H_2_O_2_ content, hormonal profiling, level of photosynthetic pigments (chlorophyll, carotenoid, and flavonoid), gaseous exchange parameters (photosynthetic rate, vapour pressure deficit, intercellular CO_2_ concentration, and intrinsic mesophyll rate), antioxidative enzymes (superoxide dismutase, polyphenol oxidase, glutathione-S transferase, and glutathione peroxidase), antioxidant assays (DPPH, ABTS, and total phenolic content), and polyphenols. Results of the present study revealed the increased H_2_O_2_ content and Cd uptake with increasing metal doses. UPLC analysis of plants showed the presence of various polyphenols. Gaseous exchange measurements were done by infrared gas analyzer (IRGA), which was negatively affected by metal treatment. In addition, LC/MS study showed the variation in the expression of plant hormones. Level of photosynthetic pigments and activities of antioxidative enzymes were altered significantly in response to metal treatment. In conclusion, the antioxidative defence system of plants got activated due to heavy metal stress, which protects the plants by scavenging free radicals.

## 1. Introduction

Heavy metals are chief environmental pollutants and their escalating toxicity causes threat for ecological and environmental reasons [1]. The principle cause of the prolonged presence of heavy metals in the environment is their nonbiodegradable nature [2]. When come in contact with the soil surface they get fervently adsorbed, followed by gradual adsorption and distribution in the soil. Plants exposed to these metals tend to accumulate them, which immensely affect their growth and development. These can not be either degraded or transformed into harmless compounds via any biological processes. Due to this they can persist in the environment for long durations [3]. Over a period of time, they enter and accumulate in the human body through food chain, which may further cause various health effects that are irreversible in nature [4].

The primary response of plants due to heavy metals stress is the production of reactive oxygen species (ROS). ROS are the partially reduced forms of atmospheric oxygen and their production is strongly regulated in normal growth conditions. Consequences of heavy metal toxicity elicit the oxidative stress in plants [5]. Phytotoxicity caused by heavy metals lead to stunted growth, leaf chlorosis, and vein necrosis and negatively affects the development of roots and leaves and also fruit quality and quantity [6, 7], [8]. Cd is enormously toxic metal, which causes reduction in stomatal density and CO_2_ conductance, and also alters the metabolic processes like respiration and nutritional status of plants. The enhanced level of ROS due to Cd stress triggers damage to DNA and leads to mutation [4].

In response to the stress, plants possess various protective mechanisms like chelation, detoxification, exclusion of metal ions through phytoremediation and activation of various stress protective proteins and osmolytes, and so forth [9–11].* Brassica juncea* L. is an amphiploid species, which belongs to Brassicaceae family. It is an oilseed crop, mainly grown as a food crop and also used for its medicinal purposes. It is one of the richest sources of iron, vitamin A, and vitamin C and also contains potassium, calcium, riboflavin thiamine, and *β*-carotene. It has antiseptic, diuretic, emetic, and rubefacient properties. It has been reported to contain antioxidants like flavonoids, carotenes, lutein, indoles, and zeaxanthin [12]. The present work was undertaken to study the effects of Cd on metal uptake, H_2_O_2_ content, hormonal profiling, level of photosynthetic pigments, gaseous exchange parameters, antioxidative enzymes, antioxidant assays, and polyphenols in* Brassica juncea* plants.

## 2. Materials and Methods

To study the effects of Cd metal on* Brassica juncea* plants, a field experiment was conducted in Botanical Garden of Guru Nanak Dev University, Amritsar, India. 20 × 20 feet area was taken for the experimentation and soil: manure in a ratio of 3 : 1 was added to it. The certified and disease-free seeds of* Brassica juncea* L. var. RLC-1 were procured from Punjab Agricultural University, Ludhiana, Punjab, India, and surface-sterilized with 0.01% mercuric chloride solution, followed by the repeated washing of sterile double distilled water (DDW). Seeds were sown in different blocks. Different treatments of Cd metal were given (0, 0.2, 0.4, and 0.6 mM Cd). Plants were then harvested after 30 days of germination to study following parameters.

### 2.1. Cadmium Accumulation

Dried plant samples were first digested by the method given by Allen et al. [13]. 0.5 g of dried plant samples was taken in digested by nitric acid : perchloric acid (2 : 1). Digested samples were cooled, filtered, and diluted up to 50 mL by DDW. The heavy metal measurement was performed with atomic absorption spectrophotometer (Shimadzu 6200). The metal content was determined by calibration with standard curve made with different concentrations of metals.

### 2.2. H_2_O_2_ Content

H_2_O_2_ content was measured by the method given by Velikova et al. [14]. To 500 mg of plant material 2 mL of TCA was added and centrifuged at 12,000 rpm for 15 minutes. Then 0.5 mL of 10 mM PPB and 1 mL of 1 M potassium iodide was added to 0.5 mL of supernatant. Absorbance was taken at 390 nm. Concentrations of H_2_O_2_ were calculated against the standard curve.

### 2.3. LC/MS Analysis of Plant Hormones

Plant samples were subjected to LC/MS in order to identify the presence of plant hormones like brassinosteroids, polyamines, auxins, abscisic acid, jasmonic acid, salicylic acid, and gibberellic acid.

*Sample Preparation*. 5 g of fresh plant sample was homogenized in 40 mL of 80% methanol. Mixture was vortexed and centrifuged. 0.2 mL of mixture was diluted to 4 mL with 80% methanol and filtered by filter papers of 0.22 micron pore size. 2 *μ*L of sample was injected for LC/MS study. Total run time of sample required in positive mode was 16 minutes and 6 minutes in negative mode. Agilent 1100 LC has been coupled with Bruker make mass spectrometer model Esquire 3000. PDA detector was used in the instrument for detecting compounds. Temperature of column was 40°C. The solvent system includes solvent A (water with 0.5% formic acid) and solvent B (methanol).

### 2.4. Photosynthetic Pigments

#### 2.4.1. Chlorophyll Content

Chlorophyll content was measured by following the method given by Arnon [15]. 1 g fresh plant tissue was homogenized by using 4 mL of 80% acetone. The homogenized material was subjected to centrifugation using Eltek cooling centrifuge for 20 minutes at 13000 rpm at a temperature of 4°C. The supernatant of plant extract was used for the analysis of chlorophyll content. The absorbance of the supernatant was taken at 645 and 663 nm.

*Calculations*. Consider:

(1) Total  Chlorophyll  Content =(Absorbance645×20.2)+(Absorbance663×8.3)  ×(V1000×W),Chlorophyll  A  content =Absorbance663×(0.058)−(Absorbance645)×0.032,Chlorophyll  B  content =Absorbance645×(0.096)  −(Absorbance663)×0.01872.

#### 2.4.2. Total Carotenoid Content

Carotenoid content was estimated by Maclachlan and Zalik [16] method. 1 g fresh plant tissue was homogenized by using 4 mL of 80% acetone. The crushed material was subjected to centrifugation using Eltek cooling centrifuge for 20 minutes at 13000 rpm at a temperature of 4°C. The supernatant from the plant extract was used for the analysis of chlorophyll content. The absorbance of the supernatant was taken at 480 and 510 nm.

*Calculations*. Consider:

(2) Total  carotenoid  content =7.6(O.D480)−1.49(O.D510)×(Vd×W×1000).

#### 2.4.3. Total Flavonoid Content

Total flavonoid content was estimated by the method given by Kim et al. [17].

*Preparation of Extract*. 1 g of fresh plant tissue was homogenized in chilled pestle and mortar using 3 mL of absolute methanol. The crushed material was then subjected to centrifugation using Eltek cooling centrifuge for 20 minutes at 13,000 rpm at a temperature of 4°C. The supernatant from the plant extract was collected for the further analysis of total flavonoid content. 1 mL of the plant extract was added to 4 mL of double distilled water. 0.3 mL of sodium nitrite (NaNO_2_) and 0.3 mL of aluminum chloride (AlCl_3_) were added to it. Then incubation was given for 5 minutes. Followed by addition of 2 mL sodium hydroxide (NaOH) pink color was developed. Then 2.4 mL of distilled water was added to it and absorbance was taken at 510 nm. 1 mg/mL of rutin was used as standard for flavonoid content determination.

### 2.5. Gaseous Exchange Parameters

Gaseous exchange parameters of plants like photosynthetic rate, vapour pressure deficit, intercellular CO_2_ concentration, and mesophyll intrinsic rate were measured with the help of infrared gas analyzer (IRGA) (Li-COR 6400). The measurement was performed within the time period 9.00–11.00 h maintaining the air temperature, air relative humidity, CO_2_ concentration, and photosynthetic photon flux density (PPFD) at 25°C, 80–90%, 400 *μ*mol mol^−1^ and 1000 *μ*mol m^−2^s^−1^, respectively.

### 2.6. Antioxidative Enzymes

*Preparation of Extract*. 1 g of harvested plant material was crushed in prechilled pestle and mortar using 3 mL of 100 mM potassium phosphate buffer (PPB) having pH 7.0. The crushed material was then subjected to centrifugation using Eltek cooling centrifuge for 20 minutes at 13,000 rpm at 4°C. The supernatant from leaf extract was collected for the various biochemical analyses.

#### 2.6.1. Superoxide Dismutase (SOD) Activity

Superoxide dismutase was estimated according to method given by Kono [18]. The method is based on the principle of the inhibitory effect of SOD on the reduction of nitroblue tetrazolium (NBT) dye by superoxide radicals, which are generated by the autooxidation of hydroxylamine hydrochloride. The reaction mixture containing 1.3 mL sodium carbonate buffer, 500 *μ*L NBT, and 100 *μ*L Triton X-100 was taken in the test cuvettes. The reaction was initiated by the addition of 100 *μ*L hydroxylamine hydrochloride. After 2 minutes, 70 *μ*L of the enzyme extract was added. The percent inhibition at the rate of NBT reduction was recorded as increase in absorbance at 540 nm.

#### 2.6.2. Polyphenol Oxidase (PPO) Activity

Activity of PPO was estimated according to the method given by Kumar and Khan [19]. Polyphenol oxidase catalyses the o-hydroxylation of monophenol (catechol) to o-diphenols and further catalyses the oxidation of o-diphenols to produce o-quinones (benzoquinones). 2.25 mL of reaction mixture contained 1 mL PPB, 0.5 mL catechol, and 0.25 mL of enzyme sample and then the reaction was held for 2 minutes at 25°C. Reaction was accomplished by adding 0.5 mL of 2.5 N H_2_SO_4_. The absorbance was read at 495 nm.

#### 2.6.3. Glutathione-S Transferase (GST) Activity

Activity of GST was measured according to the method described by Habig et al. [20]. Glutathione-S-transferase catalyzes the reaction of pharmacologically active compounds with –SH group of reduced glutathione (GSH), thereby neutralizing their electrophilic sites rendering the product more water soluble. The reaction was carried out in a total reaction mixture of 2.25 mL containing 2 mL PPB (0.2 M), pH 7.4; 100 *μ*L GSH (20 mM); 100 *μ* CDNB (20 mM); and 50 *μ*L enzyme sample. The change in absorbance at 340 nm was recorded.

#### 2.6.4. Glutathione Peroxidase (GPOX) Activity

GPOX activity was analyzed according to the method of Flohe and Gunzler [21]. GPOX stimulates the production of GSSG from GSH and H_2_O_2_. GR causes reduction of GSSH and NADPH oxidation is measured at 340 nm. In 1 mL of reaction mixture 500 *μ*L PPB, 100 *μ*L EDTA, 100 *μ*L NADPH, and 100 *μ*L H_2_O_2_ were added to a test tube. Then 50 *μ*L of enzyme extract was added to it. Decrease in absorbance due to oxidation of NADPH was measured after 1 minute.

### 2.7. Antioxidant Assays

*Preparation of Extract*. Plant samples (20 mg) were washed and oven-dried and extracted with 80% methanol for 24 hours. Extract was then filtered with Whatman number 1 filter paper. Supernatant was used for performing the following assays in ELISA reader (Biotek Synergy HT).

#### 2.7.1. DPPH Radical Scavenging Activity

This assay was performed according to the method given by Blois [22]. 0.1 mM DPPH was mixed in plant extract. The absorbance was taken at 517 nm after 20 minutes of incubation at room temperature.

*Calculations*. The inhibitory percentage of DPPH was calculated according to the following equation:

(3) $$\begin{array}{c} \%\;\text{Inhibition}=\frac{\text{Ac}-\text{As}}{\text{Ac}}\times 100. \end{array}$$

#### 2.7.2. ABTS Radical Scavenging Assay

ABTS radical scavenging assay was performed by Re et al. [23] method. Mixed 2,2′-azino-bis(3-ethylbenzthiazoline-6-sulphonic acid) and potassium persulphate in 1 : 0.5 was left for 16 hours. It was diluted with ethanol to bring the absorbance to 0.7 nm. This solution was added to the supernatant and absorbance was taken at 734 nm.

*Calculations*. The inhibitory percentage of ABTS was calculated according to the following equation:

(4) $$\begin{array}{c} \%\;\text{Inhibition}=\frac{\text{Ac}-\text{As}}{\text{Ac}}\times 100. \end{array}$$

#### 2.7.3. Total Phenolic Content

Total phenolic content was determined according to a procedure described by Singleton and Rossi [24]. In 0.4 g of dried plant material, 40 mL of 60% ethanol was added. Shaking in water was done at 60°C for 10 min. Extract was then filtered and diluted to 100 mL with 60% ethanol. From diluted plant sample, 2.5 mL was taken and rediluted with 25 mL of distilled water. 2 mL sample was mixed with 10 mL of FC reagent and then after 5 min, 2 mL of 7.5% sodium carbonate solution was added to the reaction mixture. 2 h incubation was given to the mixture. The absorbance readings were taken at 765 nm. Gallic acid was used as a reference standard.

### 2.8. UPLC Analysis of Polyphenols

*Sample Preparation*. 5 g of plant samples was homogenized in 40 mL of 80% methanol. Centrifugation was done at 13000 rpm at 4°C temperature. Then supernatant was filtered with 0.22 micron pore size filter paper and subjected to UPLC for the identification of various polyphenols like gallic acid (C_7_H_6_O_5_), epicatechin (C_15_H_14_O_6_), caffeic acid (C_9_H_8_O_4_), coumaric acid (C_9_H_8_O_3_), ellagic acid (C_14_H_6_O_8_), quercetin (C_15_H_10_O_7_), and kaempferol (C_15_H_10_O_6_) and was thinned with methanol. The plant samples were analyzed by Shimadzu UPLC Nexera system (Shimadzu, USA) coupled with photodiode array detector. C18 column (150 mm × 4.6 mm) with a pore size of 5 *μ*m is used at 25°C temperature at room temperature with a flow rate of 1 mL/min at *λ* 280 nm. The solvent system included solvent A (0.01% acetic acid in water) and solvent B (methanol). Injection volume was 5 *μ*L. Peaks were determined using software provided with Shimadzu UPLC Nexera system (USA). The calibration curves were generated by plotting concentrations versus peak areas. The detection of every compound was based on a combination of retention time and spectral similarity.

*Statistical Analysis*. Each experiment was conducted in three replicates. Data was expressed in Mean ± SE. To check the statistical significant difference between the treatments, one-way ANOVA was carried out by using Assistat version 7.7 beta.

## 3. Results

### 3.1. Metal Accumulation Study

Significant uptake of Cd metal was observed in* B. juncea *plants after 30 days of sowing (Table 1). A dose-dependent increase in uptake was found with increasing concentration of Cd. Maximum uptake (93.78 *μ*g g^−1^ DW) was noticed in 0.6 mM treated plants than in 0.4 mM (85.83 *μ*g g^−1^ DW) and 0.2 mM (78.76 *μ*g g^−1^ DW), respectively. Control plants did not show any metal uptake.

### 3.2. H_2_O_2_ Content

In present study* B. juncea *plants showed slight changes in levels of H_2_O_2_ in Cd metal treated plants when compared to untreated ones (Table 1). With the increasing dose of Cd, H_2_O_2_ content was increased in dose-dependent manner. Maximum content of H_2_O_2_ (5.93 *μ*mol g^−1^ FW) was noticed in 0.6 mM Cd treatment. Similar value of H_2_O_2_ content was recorded in 0.4 mM and 0.6 mM concentration of Cd. Level of H_2_O_2_ was found lowest in control plants (4.4 *μ*mol g^−1^ FW).

### 3.3. Hormonal Profiling by LC/MS

**Figure 1:**
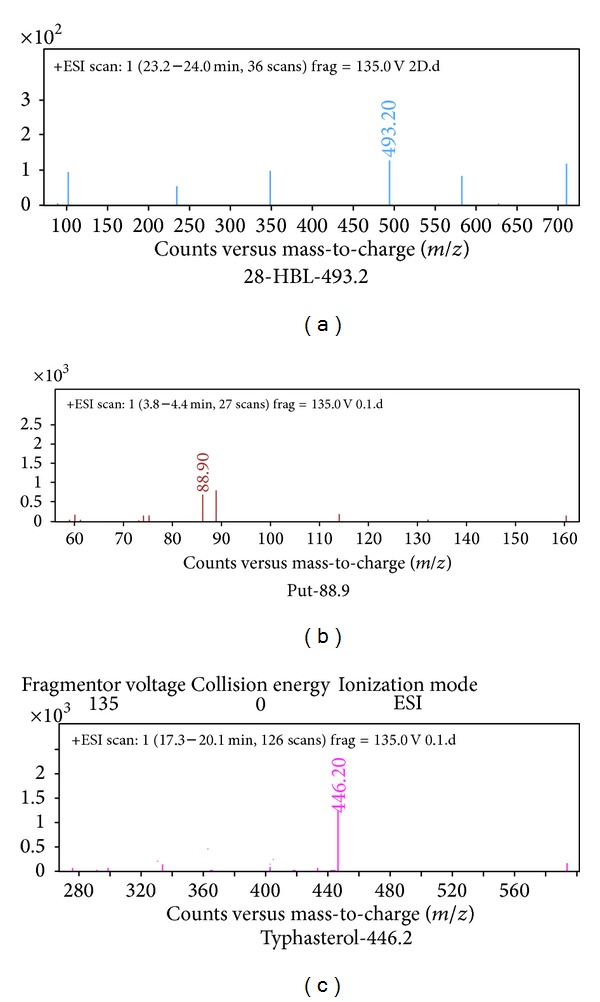
Hormonal profiling of control plants of 30-day-old* Brassica juncea*.

**Figure 2:**
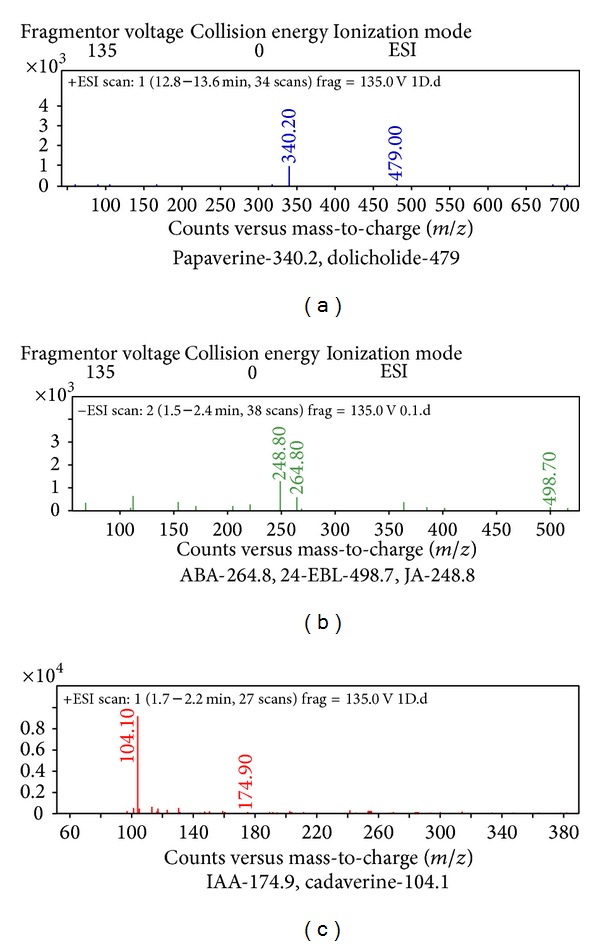
Hormonal profiling of 0.2 mM Cd treated plants of* Brassica juncea* (expression of additional hormones, 28-HBL, putrescine, and typhasterol, with respect to control).

**Figure 3:**
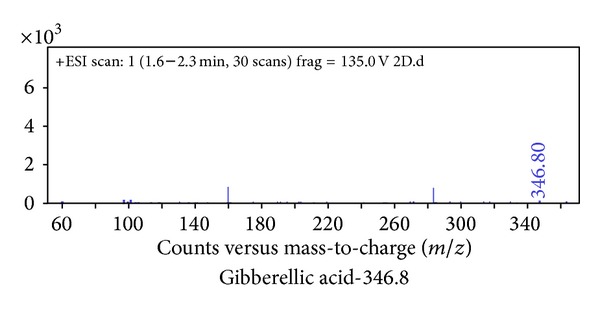
Hormonal profiling of 0.4 mM Cd treated plants of* Brassica juncea* (expression of additional hormone, gibberellic acid, with respect to other treatments).

**Figure 4:**
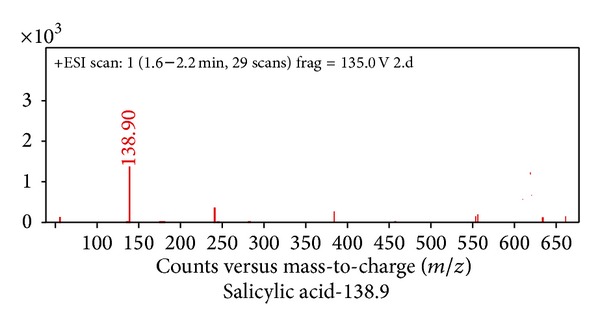
Hormonal profiling of 0.6 mM Cd treated plants of* Brassica juncea* (expression of additional hormone, salicylic acid, with respect to other treatments).

Plant hormones, namely, papaverine, dolicholide, cadaverine, abscisic acid, 24-epibrassinolide, indole 3-acetic acid, and jasmonic acid, were identified in control plants (Figure 1). Following that plant hormones got activated with enhancing doses of Cd metal. At 0.2 mM Cd typhasterol, 28-homobrassinolide and putrescine (Figure 2), at 0.4 mM Cd gibberellic acid (Figure 3) and at 0.6 mM Cd salicylic acid was also expressed (Figure 4, Table 2).

### 3.4. Photosynthetic Pigments

#### 3.4.1. Chlorophyll Content

**Figure 5:**
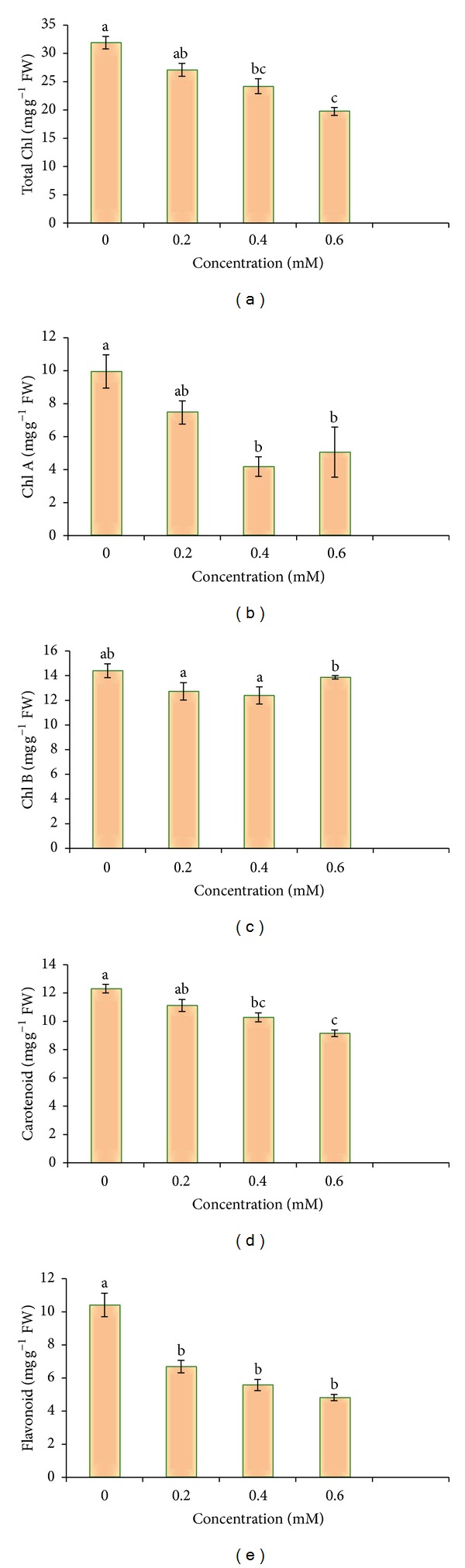
Cd metal effect on total chlorophyll, Chl A, Chl B, carotenoid, and flavonoid content of 30-day-old* B. juncea* plants.

A significant decrease in total chlorophyll content was observed in 30-day plants (Table 3, Figure 5). 1.62-fold reduction in total chlorophyll content was noticed from control (31.9 mg g^−1^ FW) plants to 0.6 mM Cd (19.72 mg g^−1^ FW). Chl A content was recorded maximum in control plants (9.85 mg g^−1^ FW) whereas 0.4 mM and 0.6 mM Cd showed a very slight variation in Chl A level, where 0.6 mM Cd contained more Chl A (4.95 mg g^−1^ FW) as compared to 0.4 mM Cd treatment (4.08 mg g^−1^ FW). Cd treatment caused very less changes in the level of Chl B. Lowest content of Chl B was recorded in the plants exposed to 0.4 mM Cd (12.39 mg g^−1^ FW) as compared to untreated control (14.4 mg g^−1^ FW). Lowest Cd toxicity was observed in the plants treated with 0.6 mM concentration, where Chl B content was highest (13.86 mg g^−1^ FW) among all treatments of Cd, which is followed by 0.2 mM Cd (12.39 mg g^−1^ FW).

#### 3.4.2. Total Carotenoid Content

*B. juncea *plants pointed out drop in the carotenoid content with the increasing concentration of Cd (Table 3, Figure 5). Carotenoid content was highest in untreated control (12.31 mg g^−1^ FW) and it got maximum decrease (9.15 mg g^−1^ FW) with the highest concentration of Cd, that is, at 0.6 mM Cd.

#### 3.4.3. Total Flavonoid Content

Results revealed the significant decrease in flavonoid content from control (10.41 mg g^−1^ FW) to 0.6 mM Cd (4.82 mg g^−1^ FW). 2.16-fold decrease in flavonoid content was noticed at 0.6 mM Cd treatment in comparison to control. 0.2 mM and 0.4 mM Cd treatment showed reduction in flavonoid level from 6.69 to 5.58 mg g^−1^ FW, respectively (Table 3, Figure 5).

### 3.5. Gaseous Exchange Parameters

#### 3.5.1. Photosynthetic Rate

**Figure 6:**
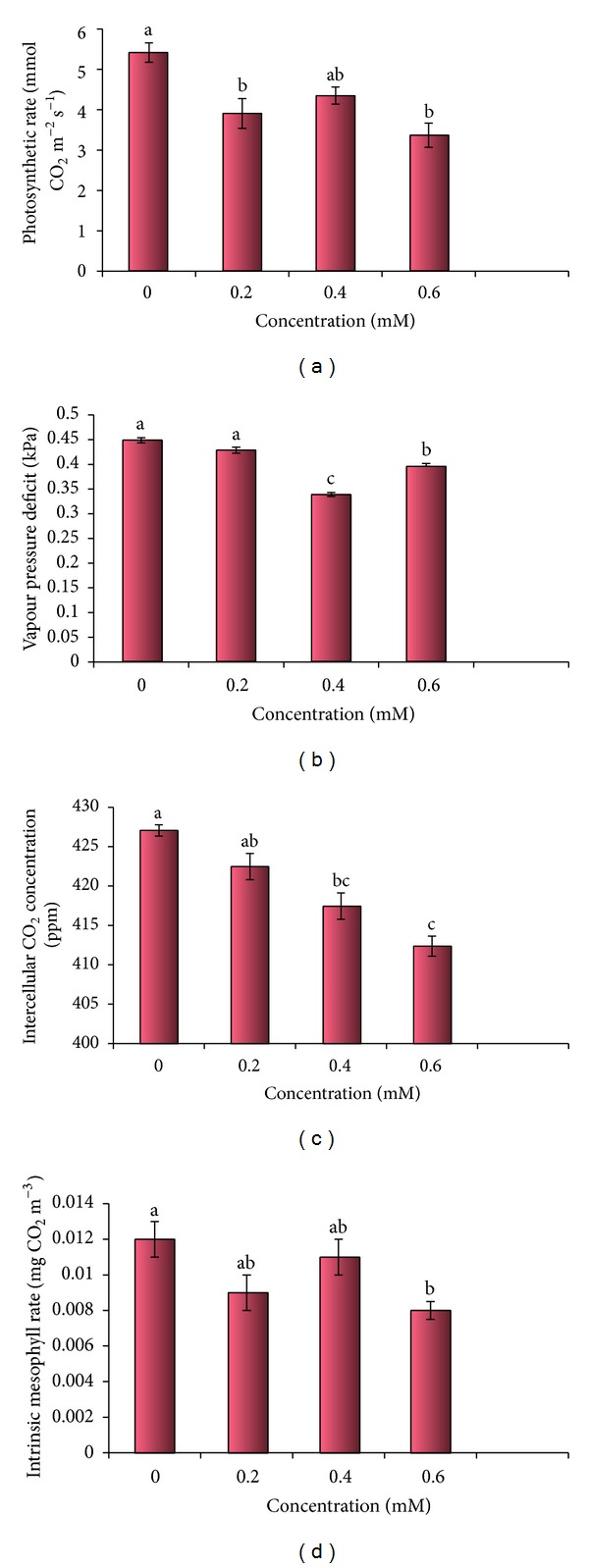
Cd metal effect on photosynthetic rate, vapour pressure deficit, intercellular CO_2_ concentration, and intrinsic mesophyll rate of 30-day-old* B. juncea* Plants.

Cd toxicity decreased the photosynthetic rate in 30-day-old plants of* B. juncea* as compared to control plants (5.42 m mol CO_2 _m^−2^ s^−1^) (Table 4, Figure 6). Minimum photosynthetic rate was noted in the plants treated with 0.6 mM of Cd (3.37 m mol CO_2 _m^−2^ s^−1^). At 0.4 mM Cd treatment (4.35 m mol CO_2 _m^−2^ s^−1^) photosynthetic rate was found to enhance as compared to 0.2 mM Cd (3.91 m mol CO_2 _m^−2^ s^−1^).

#### 3.5.2. Vapour Pressure Deficit

Cd metal toxicity altered the level of vapour pressure deficit. Vapour pressure deficit decreased with increasing Cd metal concentration (Table 4, Figure 6). Highest value was recorded in the control plants (0.45 kPa), which decreased at 0.2 mM Cd stressed plants (0.43 kPa). At 0.4 mM Cd treatment, minimum vapour pressure deficit was observed (0.34 kPa), which is lower than 0.6 mM Cd treatment (0.4 kPa).

#### 3.5.3. Intercellular CO_2_ Concentration (Ci)

A continuous decline was noticed in the intercellular CO_2_ concentration, when Cd treatment was given to plants (Table 4, Figure 6). Lowest value was observed in 0.6 mM Cd stressed plants (412.37 ppm). Decrease in Ci value was recorded from control (427.07 ppm) to 0.4 mM Cd (417.44 ppm).

#### 3.5.4. Intrinsic Mesophyll Rate

Very small variation was noticed in intrinsic mesophyll rate. Maximum value was possessed by control plants (0.012 mmol CO_2_ m^−3^). With metal treatment highest mesophyll rate was recorded in 0.4 mM Cd treatment (0.011 mmol CO_2_ m^−3^), which was slightly lower than control. 0.2 mM (0.009 mmol CO_2_ m^−3^) and 0.6 mM Cd (0.008 mmol CO_2_ m^−3^) stress showed nearly similar intrinsic mesophyll rate (Table 4, Figure 6).

### 3.6. Antioxidative Enzymes

**Figure 7:**
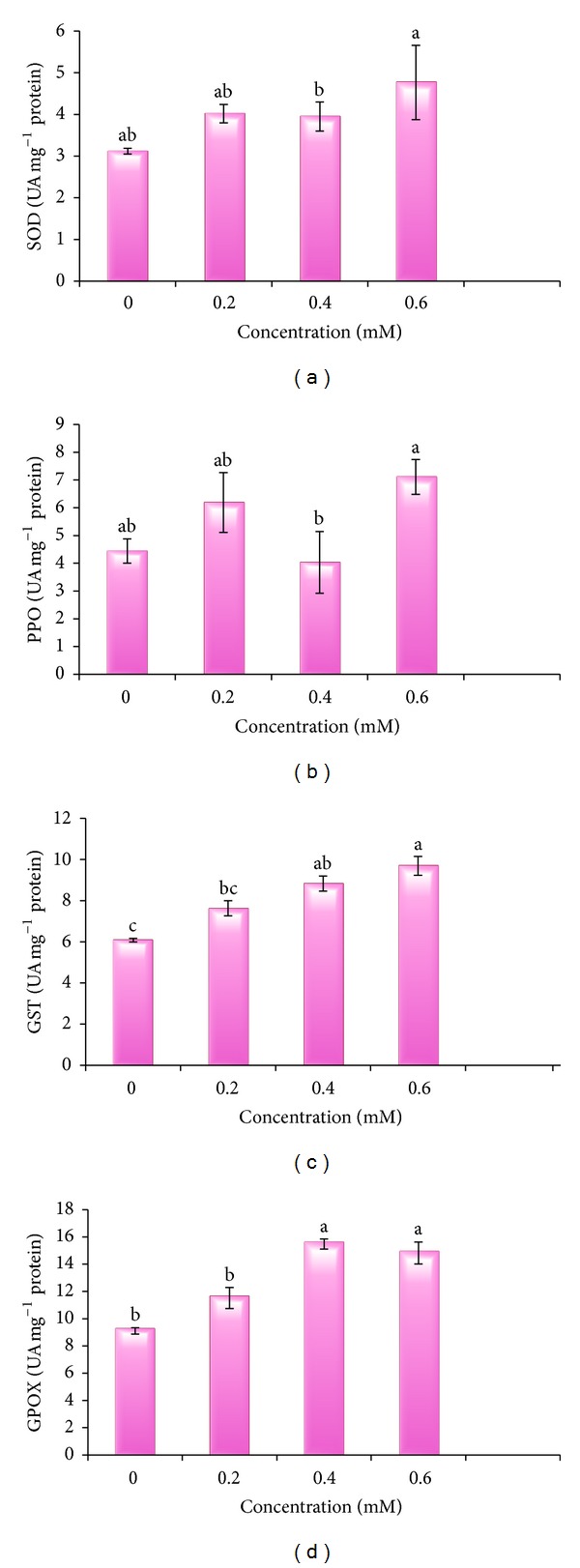
Cd metal effect on activities of SOD, PPO, GST, and GPOX of 30-day-old* B. juncea* plants.

Activities of all the enzymes SOD, PPO, GST, and GPOX were enhanced with the increased dose of Cd compared to control plants (Table 5, Figure 7). A continuous increase in the activity of GST was observed. Minimum activity of enzyme was measured in control plants, that is, 6.08 UA mg^−1^ protein. Cd toxicity enhanced the activity of GST from 0.2 mM (7.63 UA mg^−1^ protein) to 0.6 mM Cd (9.69 UA mg^−1^ protein). Highest metal treatment showed highest activity of enzyme. Results revealed the maximum GPOX activity at 0.4 mM Cd treated plants as compared to untreated control (9.1 UA mg^−1^ protein). Activity of GPOX enzyme at 0.2 and 0.6 mM Cd was 11.52 and 14.83 UA mg^−1^ protein, respectively. Slight variations in activities of SOD and PPO enzymes were noticed in present study. Untreated control plants showed the lowest enzymes activities (3.12 and 4.44 UA mg^−1^ protein, resp.). Then got increase in the activities from control to 0.2 mM Cd stressed plants. An increase in SOD activity from 3.12 to 4.02 UA mg^−1^ protein and from 4.44 to 6.19 UA mg^−1^ protein for PPO was observed. Activities of enzymes were again inhibited at 0.4 mM Cd treated plants. At 0.4 mM Cd treatment, activities of SOD and PPO decreased to 3.95 and 4.03 UA mg^−1^ protein, respectively, in comparison to 0.2 mM Cd. Further, 0.6 mM Cd toxicity caused rise in enzyme activities from 3.12 to 3.95 UA mg^−1^ protein (SOD) and from 4.44 to 4.03 UA mg^−1^ protein (PPO).

### 3.7. Antioxidant Assays

#### 3.7.1. DPPH

**Figure 8:**
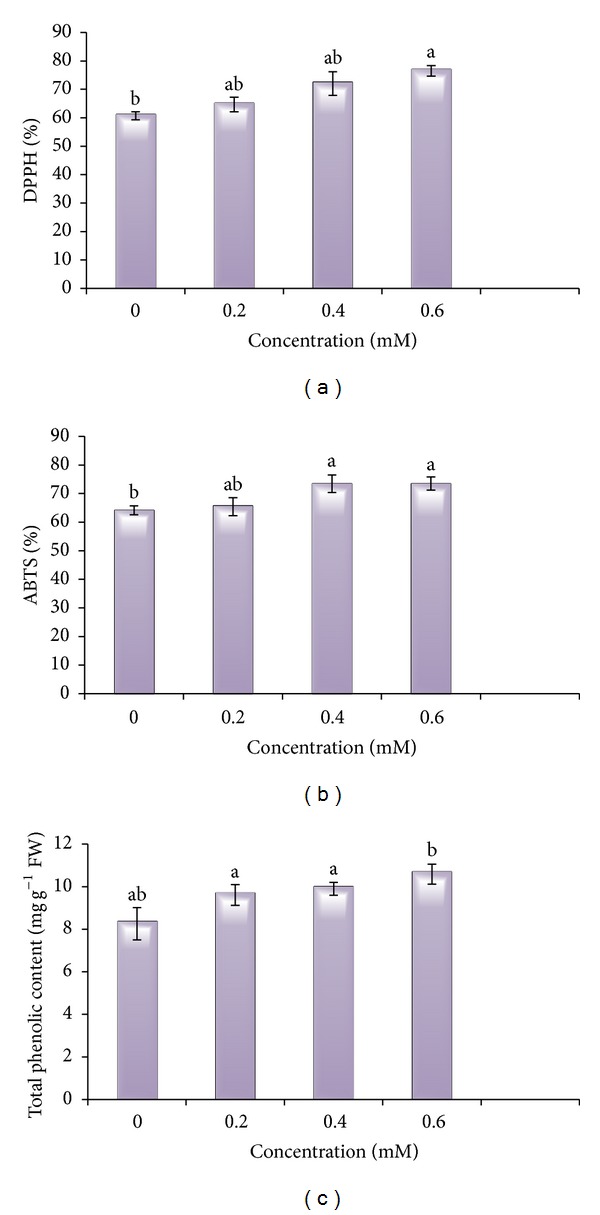
Cd metal effect on scavenging activities of DPPH, ABTS, and total phenolic content of 30-day-old* B. juncea* plants.

Results revealed the increase in scavenging of DPPH radical by Cd metal treated plants in comparison to control (60.69%). DPPH inhibition was enhanced maximum at 0.6 mM stressed plants (76.55%). In 0.2 mM Cd and 0.4 mM Cd stressed plants, inhibition of DPPH radical was observed (64.66 and 72.02%, resp.) (Table 6, Figure 8).

#### 3.7.2. ABTS

In present study, 0.6 mM Cd (73.55%) was found to possess maximum potential to scavenge ABTS as compared to control (64.11%) (Table 6, Figure 8). Very less difference in scavenging potential was observed between 0.4 mM (73.46%) and 0.6 mm Cd treatment (73.55%).

#### 3.7.3. Total Phenolic Content

With increasing Cd toxicity, total phenolic content also increased in dose-dependent manner (Table 6, Figure 8). Phenol content was found maximum in 0.6 mM Cd stressed plants, that is, 10.59 mg g^−1^ FW, in comparison to control plants (8.26 mg g^−1^ FW). An increase was also observed from 8.26 to 9.61 (0.2 mM) and 9.9 mg g^−1^ FW (0.4 mM Cd).

### 3.8. UPLC Analysis of Polyphenols

**Figure 9:**
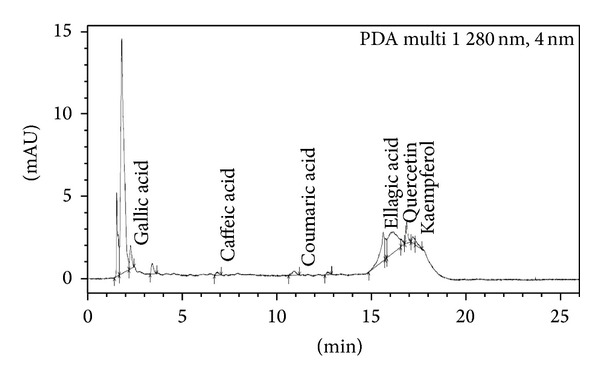
UPLC chromatograph of control plants of 30-day-old* Brassica juncea*.

**Figure 10:**
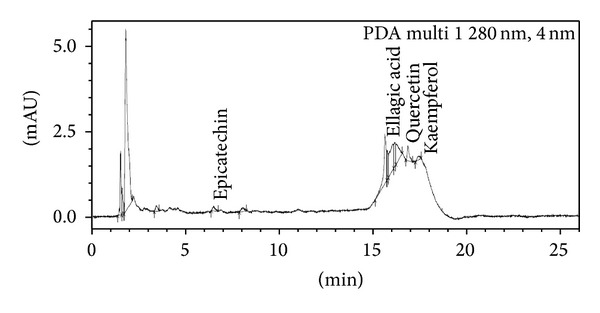
UPLC chromatograph of 0.2 mM Cd treated 30-day-old plants of* Brassica juncea*.

**Figure 11:**
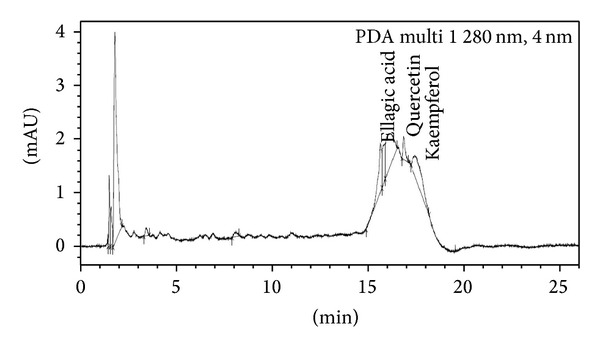
UPLC chromatograph of 0.4 mM Cd treated 30-day-old plants of* Brassica juncea*.

**Figure 12:**
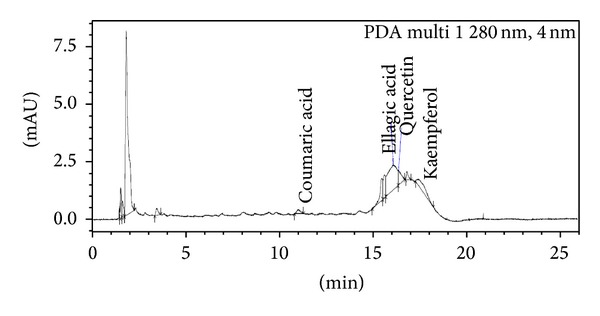
UPLC chromatograph of 0.6 mM Cd treated 30-day-old plants of* Brassica juncea*.

Chromatograph showed that gallic acid, caffeic acid, coumaric acid, ellagic acid, quercetin, and kaempferol were identified in the present study (Figure 9, Table 7). In 0.2 mM Cd stress, ellagic acid, quercetin, and kaempferol were expressed and one additional polyphenol, namely, epicatechin, was also observed in comparison to control (Figure 10). Distinct peaks of quercetin and kaempferol showed their more expression in 0.4 mM and 0.6 mM Cd stressed plants as compared to untreated control (Figures 11 and 12, resp.). Percentage of the phenolic compounds is given in Table 6.

## 4. Discussion

Heavy metal stress has become a foremost focal point due to the increased environmental pollution. Metals are nonbiodegradable, so they often cause lethal biological effects [25]. Heavy metals lead to the formation of oxidants/free radicals. It is the primary response of plants exposed to stress. Reduced forms of atmospheric oxygen (O_2_) are the intermediates of ROS. Generation of ROS results from the excitation of O_2_, which forms the singlet oxygen (^1^O_2_). These intermediates are formed from the transfer of electrons, which generate hydrogen peroxide (H_2_O_2_), superoxide radical (O_2_^•−^), and hydroxyl radical (HO^•−^) [26]. Present study also showed the increased level of H_2_O_2_ with increasing Cd doses. It may be due to the destabilization of membrane in plants with increasing metal stress [27] as the plants were found to accumulate more Cd with enhancing its doses. Production of ROS occurs due to oxidative stress or through Haber-Weiss reactions [5]. Various deleterious effects of free radicals collectively cause oxidative stress. Serious imbalance is caused in antioxidative system due to the production of reactive oxygen species (ROS) and reactive nitrogen species (RNS) during oxidative stress.

Plants possess certain stress protective mechanisms such as antioxidative defence systems which include plant growth regulators and antioxidative enzymes [28]. Antioxidative enzymes like SOD, POD, PPO, and GPOX help in the scavenging of free radicals. Certain stress protective proteins like heat shock proteins protect plants against oxidative damage [29]. Due to heavy metal toxicity, several types of defence responses are produced in plants, but their action depend upon the doses, type of plant species, and so forth [30]. Ability of plants to ameliorate the heavy metal toxicity or to bear the stress makes them survive in those conditions [31]. Exposure of heavy metals activates the antioxidative defence system. Similarly in the present work, increased activities of SOD, PPO, GST, and GPOX enzymes were stimulated with metal treatment and thus helped in the scavenging of free radicals like DPPH. These results are in coherence with the findings of Doganlar et al. [32]. Antioxidative potential of plant was enhanced in dose-dependent manner.

Another mechanism of defense in plants involves the secondary metabolites and PGRs. Plant hormones like auxins, abscisic acid, brassinosteroids, and polyamines regulate metabolic processes related to plant growth and development and they have also been found to work as stress protectants by scavenging the reactive oxygen species [33]. These hormones activate the antioxidative defence system of plants exposed to stress and thus help in amelioration of stress [34, 35]. Similarly, in present study, hormones were much expressed in metal treated plants. These results were supported by the findings of Groppa et al. [36, 37] where putrescine biosynthesis was found to enhance under Cu and Cd stress in sunflower discs. The rise in putrescine synthesis was due to increased activities of ornithine decarboxylase (ODC) and arginine decarboxylase (ADC) enzymes, which leads to synthesis of hormone. Similarly, Atici et al. [38] recorded significant rise in the endogenous levels of ABA in the seeds of chick pea exposed to Zn and Pb stress. The present work was also in coherence with the findings of Munzuroglu et al. [39], where Hg, Cu, and Cd toxicity caused significant enhancement in the ABA in wheat seeds.

Level of photosynthetic pigments was recorded to decrease in the present investigation with increasing Cd doses. Similar, findings were reported in tomato, mustard, and garden cress [40–42] when exposed to Cd metal. It may be due to the fact that Cd causes inhibition of Fe and leads to chlorosis of leaves, thus negatively affecting chlorophyll metabolism [43]. Micronutrients are also degraded by the toxicity of heavy metals, which are required for the growth and development of plants. Consequently, level of pigments falls under metal stress [44]. This is also one of major reasons, which lead to photosynthesis impairment. Similar results were obtained from the present work, where fall in gaseous exchange measurements was observed. These results are in coherence with the findings of Januškaitienė [45], where gaseous exchange parameters like photosynthetic rate, intercellular CO_2_ concentration, and so forth decreased with Cd metal stress in pea plants.

## 5. Conclusion

Cd is one of the most toxic heavy metals, which increases the production of ROS like H_2_O_2_. Metabolic activities are altered by Cd stress. Various defence mechanisms of* Brassica juncea *plants got activated to combat the stress, like antioxidative defence system and hormonal level. Thus, the plants' own defensive strategies provide protection to plants from oxidative stress generated by Cd.

## Figures and Tables

**Table 1:** Effect of Cd metal on Cd uptake and H_2_O_2_ content of 30-day-old *B.  juncea *plants.

Days of harvesting	Treatments							
	Cd uptake (*μ*g g^−1^ DW)				H_2_O_2_ content (*μ*mol g^−1^ FW)			
	0 mM	0.2 mM	0.4 mM	0.6 mM	0 mM	0.2 mM	0.4 mM	0.6 mM
30 days	0.0 ± 0.0^c^	78.76 ± 2.65^b^	85.83 ± 1.61^b^	93.78 ± 1.29^a^	4.4 ± 0.25^b^	4.59 ± 0.30^b^	5.93 ± 0.42^a^	5.93 ± 0.06^a^

Data presented in mean ± SE. Different letters (a, b, & c) within various concentrations of Cd (0, 0.2, and 0.4 mM) are significantly different (Fisher LSD *post hoc test, P* ≤ 0.05).

**Table 2:** Hormonal profiling of *Brassica juncea *plants exposed to different concentrations of Cd.

S. number	Treatments	Hormones
1	Control	Papaverine, dolicholide, abscisic acid (ABA), 24-epibrassinolide (EBL), jasmonic acid (JA), indole-3-acetic acid (IAA), and cadaverine
2	0.2 mM Cd	Papaverine, dolicholide, ABA, JA, IAA, cadaverine, 28-homobrassinolide (HBL), putrescine (Put), and typhasterol
3	0.4 mM Cd	Papaverine, dolicholide, ABA, 24-EBL, JA, IAA, cadaverine, 28-HBL, Put, typhasterol, and gibberellic acid
4	0.6 mM Cd	Papaverine, dolicholide, ABA, 24-EBL, JA, IAA, cadaverine, 28-HBL, Put, typhasterol, gibberellic acid, and salicylic acid

**Table 3:** Effect of Cd metal on total chlorophyll, Chl A, Chl B, carotenoid and flavonoid content of 30- day-old *B.  juncea *plants.

Treatments	Total Chl (mg g^−1^ FW)	Chl A (mg g^−1^ FW)	Chl B (mg g^−1^ FW)	Carotenoid (mg g^−1^ FW)	Flavonoid (mg g^−1^ FW)
0.0 mM	31.9 ± 1.11^a^	9.85 ± 1.01^a^	14.4 ± 0.56^ab^	12.31 ± 0.31^a^	10.41 ± 0.71^a^
0.2 mM	27.09 ± 1.14^ab^	7.36 ± 0.71^ab^	12.73 ± 0.71^a^	11.12 ± 0.42^ab^	6.69 ± 0.38^b^
0.4 mM	24.21 ± 1.31^bc^	4.08 ± 0.59^b^	12.39 ± 0.70^a^	10.28 ± 0.32^bc^	5.58 ± 0.34^b^
0.6 mM	19.72 ± 0.70^c^	4.95 ± 1.52^b^	13.86 ± 0.14^b^	9.15 ± 0.23^c^	4.82 ± 0.19^b^

**Table 4:** Effect of Cd metal on photosynthetic rate, vapour pressure deficit, intercellular CO_2_ concentration, and intrinsic mesophyll rate of 30-day-old *B.  juncea *plants.

Treatments	Photosynthetic rate (mmol CO_2_ m^−2^s^−1^)	Vapour pressure deficit (kPa)	Intercellular CO_2_ concentration (ppm)	Intrinsic mesophyll rate (mmol CO_2_ m^−3^)
0.0 mM	5.42 ± 0.24^a^	0.45 ± 0.005^a^	427.07 ± 0.72^a^	0.012 ± 0.001^a^
0.2 mM	3.91 ± 0.37^b^	0.43 ± 0.006^a^	422.48 ± 1.68^ab^	0.009 ± 0.001^ab^
0.4 mM	4.35 ± 0.21^ab^	0.34 ± 0.004^c^	417.44 ± 1.67^bc^	0.011 ± 0.001^ab^
0.6 mM	3.37 ± 0.3^b^	0.4 ± 0.003^b^	412.37 ± 1.26^c^	0.008 ± 0.0005^b^

**Table 5:** Effect of Cd metal on specific activities of SOD, PPO, GST, and GPOX of 30-day-old *B.  juncea *plants.

Treatments	SOD (UA mg^−1^ protein)	PPO (UA mg^−1^ protein)	GST (UA mg^−1^ protein)	GPOX (UA mg^−1^ protein)
0.0 mM	3.12 ± 0.07^ab^	4.44 ± 0.44^ab^	6.08 ± 0.09^c^	9.1 ± 0.23^b^
0.2 mM	4.02 ± 0.22^ab^	6.19 ± 1.08^ab^	7.63 ± 0.37^bc^	11.52 ± 0.77^b^
0.4 mM	3.95 ± 0.35^b^	4.03 ± 1.11^b^	8.83 ± 0.37^ab^	15.49 ± 0.37^a^
0.6 mM	4.77 ± 0.89^a^	7.11 ± 0.63^a^	9.69 ± 0.46^a^	14.83 ± 0.80^a^

**Table 6:** Effect of Cd metal on scavenging activities of DPPH, ABTS, and total phenolic content of 30- day-old *B.  juncea* plants.

Treatments	DPPH (%)	ABTS (%)	Total phenolic content (mg g^−1^ FW)
0.0 mM	60.69 ± 1.42^b^	64.11 ± 1.57^b^	8.26 ± 0.76^ab^
0.2 mM	64.66 ± 2.59^ab^	65.44 ± 3.15^ab^	9.61 ± 0.49^a^
0.4 mM	72.02 ± 4.17^ab^	73.46 ± 3.08^a^	9.9 ± 0.31^a^
0.6 mM	76.55 ± 1.84^a^	73.55 ± 2.29^a^	10.59 ± 0.47^b^

**Table 7:** Concentrations of phenolic compounds of 30-day-old *Brassica juncea *plants treated with Cd stress.

S. number	Polyphenolic compound	Percentage			
		Control	0.2 mM Cd	0.4 mM Cd	0.6 mM Cd
1	Gallic acid	0.743	—	—	—
2	Caffeic acid	0.143	—	—	—
3	Coumaric acid	0.138	—	—	0.084
4	Ellagic acid	5.868	2.914	2.510	2.045
5	Quercetin	0.437	0.473	0.468	0.948
6	Kaempferol	1.561	0.487	6.285	4.700
7	Epicatechin	—	0.338	—	—
